# Social network and dominance hierarchy analyses at Chimpanzee Sanctuary Northwest

**DOI:** 10.1371/journal.pone.0191898

**Published:** 2018-02-14

**Authors:** Jake A. Funkhouser, Jessica A. Mayhew, John B. Mulcahy

**Affiliations:** 1 Primate Behavior and Ecology Program, Central Washington University, Ellensburg, Washington, United States of America; 2 Department of Anthropology and Museum Studies, Central Washington University, Ellensburg, Washington, United States of America; 3 Chimpanzee Sanctuary Northwest, Cle Elum, Washington, United States of America; University of Portsmouth, UNITED KINGDOM

## Abstract

Different aspects of sociality bear considerable weight on the individual- and group-level welfare of captive nonhuman primates. Social Network Analysis (SNA) is a useful tool for gaining a holistic understanding of the dynamic social relationships of captive primate groups. Gaining a greater understanding of captive chimpanzees through investigations of centrality, preferred and avoided relationships, dominance hierarchy, and social network diagrams can be useful in advising current management practices in sanctuaries and other captive settings. In this study, we investigated the dyadic social relationships, group-level social networks, and dominance hierarchy of seven chimpanzees (*Pan troglodytes*) at Chimpanzee Sanctuary Northwest. We used focal-animal and instantaneous scan sampling to collect 106.75 total hours of associative, affiliative, and agonistic data from June to September 2016. We analyzed our data using SOCPROG to derive dominance hierarchies and network statistics, and we diagrammed the group’s social networks in NetDraw. Three individuals were most central in the grooming network, while two others had little connection. Through agonistic networks, we found that group members reciprocally exhibited agonism, and the group’s dominance hierarchy was statistically non-linear. One chimpanzee emerged as the most dominant through agonism but was least connected to other group members across affiliative networks. Our results indicate that the conventional methods used to calculate individuals’ dominance rank may be inadequate to wholly depict a group’s social relationships in captive sanctuary populations. Our results have an applied component that can aid sanctuary staff in a variety of ways to best ensure the improvement of group welfare.

## Introduction

In 2015, the United States Fish and Wildlife Services declared captive chimpanzees (*Pan troglodytes*) endangered under the Endangered Species Act, effectively ending invasive biomedical research on chimpanzees in the United States [1]. Consequently, hundreds of chimpanzees in laboratories are in need of relocation to sanctuary for retirement, which necessitates research to broadly understand chimpanzees’ captive needs as well as the needs of this specific population [2–3]. In the wild, chimpanzees occupy wide ranges to obtain resources and are challenged socially as they navigate complex fission-fusion societies and strict dominance hierarchies [4–5]. Similar social and ecological conditions are difficult to recreate in captivity and therefore present many challenges for sanctuary staff and managers tasked with ensuring species-typical behavior and maximizing welfare. Also, any sanctuary-living chimpanzee groups are composed differently than their wild counterparts. It is not abnormal for such laboratory-retired captive groups to contain relatively few individuals, all of similar age, with skewed sex ratios (mostly male or mostly female), unique or uncertain rearing histories, no genetic relatedness, and long-term membership. Given that early rearing greatly impacts the behavior and personality of adult chimpanzees (e.g., [6–8]) and many approximations of social status have been assumed through measures of kinship [9], wild-observed predictors of social relationship value, directionality, or overall social structure cannot be readily applied to sanctuary-living groups. Thus, it is important to further our understanding of how to assess group structure in unique captive groups and extrapolate as to how these results can be applied by sanctuary managers to improve captive welfare.

Social Network Analysis (SNA) has emerged as a promising tool to assess group social structure using multiple behavioral measures. SNA can be broadly defined as the use of matrix-based data to analyze individual dyadic or group-level social interactions through any one or multiple network-based descriptive and statistical analyses [10]. SNA can be used to derive dyadic relationship indexes and network statistics for each individual; identify clusters, subgroups, isolated individuals, and preferred or avoided dyads; and diagram the group’s social network [11–12]. SNA is useful for characterizing multifaceted social relationships (such as those of chimpanzees) because it allows for analyses of different relationship measures and (depending on the question at hand) can be performed at multiple levels, from the individual to the larger population [13]. Some posit that a greater understanding of social system complexity will only come about when we investigate social relationships beyond the dyad [14]. SNA offers a framework to identify how dyadic relationships relate to the larger social structure, characterize group- or population-wide social relationships in a comparable way, and analyze relational links between all group members (e.g., [10, 15]). SNA can also compare or combine different social relationship metrics to aid in accurately examining and illustrating the quality of nonhuman primate social relationships [9, 16–17]. In chimpanzees, specifically, social relationships have been framed through a variety of behavioral measures, including dyadic grooming [18], polyadic grooming or gatherings [19], varying degrees of proximity [20], rest in contact [21], social play [22], gestural communication [23], as well as a number of metrics (e.g., rates, counts, durations).

Similarly, calculating social dominance hierarchies in *Pan* can be equally challenging. Many researchers have constructed linear dominance hierarchies based on submissive behaviors [24–27] or attack-retreat/win-loss interactions [28–29]. Others have assembled dominance hierarchies of only one sex (typically males) based on high-middle-low classifications of dominance positions [30]. Certain SNA statistical programs can analyze dominance while also offering a network approach addressing the flexible and dynamic nature of dominance relationships [13]. Additionally, the presence of unknown (or null) agonistic relationships can affect conventional analyses of dominance [31]. Therefore, a network approach is useful with a dataset containing limited interactions, particularly when characterizing a group’s agonistic network globally without underlying assumptions of linearity [32]. Characterizing a network in a global fashion helps to construct a holistic understanding of captive primate social relationships and, through this increased understanding, can contribute to the universal goal of continued, improved captive welfare. The task of ensuring positive welfare states (e.g., [33–35]) in captive individuals and identifying agency in social relationships is challenging because of the complexity of nonhuman primate social behavior (both within and between species).

SNA has been used to assess the welfare of human-managed animal groups, including horses, captive brown bears, laying hens, and veal calves [36]. Managing the social network of a captive animal population as well as the stability and structure of agonistic and affiliative networks is vital to manage deleterious aggression, morbidity, and mortality, thereby ensuring adequate captive welfare [13, 37]. SNA can help identify individuals who asymmetrically participate in aggression and guide management in modifying group composition (e.g., remove/introduce individuals, divide large subgroups if deemed appropriate) to achieve network stability [2, 10, 37]. In captive chimpanzees, increasing affiliative network cohesion (i.e., increasing grooming reciprocity) has been demonstrated to decrease a group’s agonism, behavioral indicators of stress, and social tension [38–40]. The use of SNA to increase our understanding of social complexity has demonstrated that highly central individuals (in affiliative networks) are important to maintain group cohesion; these central individuals might aid in the successful integration of new individuals and influence others’ individual welfare by decreasing social tension and individual levels of stress [11, 37, 40–41]. Measures of network centrality and the identification of significant subgroups (or clusters) through SNA are important to consider when introducing new individuals or determining who to transfer [10, 11, 40, 42]. However, SNA research with sanctuary-living chimpanzees is limited, specifically regarding biomedical individuals with heterogeneous rearing histories. Conversely, analyses of captive primate dominance hierarchies are recurrent in the literature but generate few generalizable results between groups and utilize multiple methods to calculate dominance ranks. Furthermore, using conventional approaches to analyze dominance in the sanctuary setting may be difficult because aggression rates may be low [43–44], sex ratios heavily skewed, and differing husbandry practices by caretakers may influence group social dynamics [45].

To examine sanctuary-specific chimpanzee social group structure, we used SNA to calculate social network statistics for each individual (i.e., affinity, strength, reach, eigenvector centrality), identify significant subgroups or clusters (i.e., divisions of modularity and hierarchical clusters), and construct network diagrams (i.e., principal coordinate analyses). We also examined the dominance hierarchy of a sanctuary-living chimpanzee group using conventional methods: modified David’s scores [46], I & IS [47], Brown’s method [48], and hierarchical clusters [30]. In doing so, we aim to better advise captive management and welfare decisions for sanctuary-living chimpanzee groups with varied rearing and biomedical histories. In turn, other U.S. sanctuaries and captive primate institutions can use this SNA case study to aid in the management of their own groups.

## Methods

### Study subjects and site

We observed one group of captive chimpanzees (*Pan troglodytes*) at Chimpanzee Sanctuary Northwest (CSNW) in Cle Elum, WA. This group is composed of one male and six females (*N* = 7), ages 33 to 43 years (Table 1) with no genetic relatedness. Little is known about each individual’s specific early life history; however, their experiences range from any combination of living in human homes as pets, in the entertainment industry, as research subjects, or breeding individuals in biomedical laboratories. Similarly, the chimpanzees have unknown origins but range from wild-caught to laboratory-born. All seven chimpanzees retired to CSNW from a biomedical research facility in June 2008. This group’s members have been exclusively housed together since arriving at the sanctuary. It is presumed they were housed together for four years prior to arrival, but it is unclear whether there were additional individuals living with them during that time.

**Table 1:** Chimpanzee demographics.

Name	Abbreviation	Sex	Estimated Age
Annie	Ann	F	42
Burrito	Bur	M	33
Foxie	Fox	F	40
Jamie	Jam	F	38
Jody	Jod	F	41
Missy	Mis	F	41
Negra	Neg	F	43

Age is provided as an estimate (in years) when data was collected from June to September of 2016.

During data collection, the chimpanzees had variable access to a total of seven conjoined enclosure spaces: three small indoor rooms (~9.5 m^2^ each), one slightly larger indoor room (~13 m^2^), one large two-story indoor room (~111 m^2^), one indoor-outdoor space (caged walls with solid roof and bark substrate) with climbing structures (~56 m^2^), and one large open-topped outdoor space (electric-fenced and earth substrate) with multiple climbing structures (~1 ha). The chimpanzees were provided three meals per day consisting of various fruits, vegetables, and manufactured primate “chow” (breakfast: 10:00; lunch: 13:00; dinner: 16:30) either served by a caregiver or forage-style. The chimpanzees also had access to water *ad libitum* and were provided object enrichment each morning and food-puzzle enrichment each evening.

### Data collection

J.A.F collected behavioral data on three randomly assigned days per week from June 16 to September 9, 2016 (*N* = 31 days) using a combination of 15-minute focal-animal and instantaneous scan sampling [49]. For both sampling methods, we used the same ethogram (modified from the AZA [50]) to operationally define and categorize behaviors (Table 2). We focal-followed each individual for 15 minutes twice per day in a random sequence. We observed each individual once before progressing to the second cycle of randomly ordered observations. Instantaneous scan samples were collected between each focal-animal sample. During scan sampling, we recorded each chimpanzee’s behavior and proximate individuals as encountered progressing across the facility from south to north. When multiple chimpanzees were on the same longitudinal line, we observed them from west to east. Inter-observer reliability was calculated between J.A.F and an independent coder during preliminary data collection. We collected behavioral data on an iPad (2nd generation) using Animal Behaviour Pro [51] and later transcribed the data into Microsoft Excel. Our research complied with the protocol approved by Central Washington University’s Institutional Animal Care and Use Committee (protocol no. A041604), this reach also followed the American Society of Primatologists’ Principles for the Ethical Treatment of Primates.

**Table 2:** Behavioral ethogram.

Behavior	Description
*Association*	
Proximity	Any individual within the subject’s reach. Proximity during locomotion refers to an individual within the subject’s reach and moving in the same direction.
*Affiliation*	
Allogroom	Picking through hair or at skin of another individual, removing debris with hands and/or mouth. Does not include pulling hair; the actor receives no grooming at the same time.
Play	Non-aggressive interactions involving two or more chimpanzees. Never accompanied by pilo-erection or agonism; may be accompanied by play-face and/or laughing. Includes rough-and-tumble play, quiet play, object play, self play, and social play initiation.
Simultaneous Groom	Picking through hair or at skin of another individual, removing debris with hands and/or mouth, while the other individual returns the same behaviors at the same time. Does not include pulling hair.
Other Affiliation	Any other affiliative behavior; may or may not involve contact.
*Agonism*	
Displace	Approaching and taking the physical space of another individual.
Display	Aggressive behavior without any clear and identifiable recipient. May include pilo-erection, and such behaviors as beating on or moving inanimate objects, stomping, slapping, swaying, hooting, chest-beating, or running.
Fight	Reciprocal contact aggression that continues into a state.
Submission	Includes crouching, bobbing, fleeing, avoiding, fear grimacing, bared teeth creaming, and pant grunting.
Threat	Aggressive behaviors directed to another individual that do not include any physical contact. Includes lunge and rush.
Other Aggression	Aggressive behaviors that must involve some physical contact between individuals. Includes, wrestling, lunge, hit, grab, bite, and scratch. May include pilo-erection. Also including any other behaviors perceived as agonistic in nature.

This ethogram was modified from the Association of Zoos and Aquariums [50].

### Analyses

We performed all social and hierarchical structure analyses in SOCPROG [52] using *allogrooming* (asymmetric interactions) durations collected during focal samples, *occurrences of dyads in proximity* (symmetric associations) collected during scan sampling, and *agonistic events* (asymmetric interactions) compiled across both collection methods. Because we considered each agonistic or submissive encounter as a single occurrence, we summed agonistic interactions across both sampling methods to increase the size of the dataset. We collected directional allogrooming durations (seconds) and represented the data in a weighted edgelist (a type of SNA data file); to do this, we transformed the data so that each second of a grooming bout was entered as a single event so that SOCPROG would compile the total number of seconds spent grooming for each dyad (e.g., a 14-second bout was entered as 14 different events but the dyad’s score remained 14). Because this investigation aims, broadly, to describe the social structure of this group through multiple tests of SNA, we’ve followed Whitehead’s suggestion of collecting more than 30 days of data to construct an informative study [53]. However, caution is necessary when planning studies that utilize SNA as minimum data requirements, statistical assumptions, and specific test requirements vary based on a number of factors (e.g., dyadic or global questions of social structure, number of individuals, social differentiation, species gregariousness, etc.). A full review of SNA and these statistical considerations can be found in Whitehead [52–53].

#### Dyadic indexes

Using *simple ratio* methods, we calculated association and interaction indexes for all dyads across directional (asymmetric) agonistic and allogrooming matrixes and unidirectional (symmetric) associative matrixes [53]. We then used these matrixes to construct a *hierarchical cluster analysis* in SOCPROG to illustrate the clustering of relationships between individuals (three or more are required). This dendrogram was accompanied by a cophenetic correlation coefficient (CCC), where coefficients greater than 0.80 indicate an appropriate visual representation of the association indexes [54]. We later drew *principal coordinate analyses* in NetDraw [55] to descriptively illustrate the relationships and observed interactions between chimpanzees. These analyses plot individuals (two or more are required) with strong associations nearest one another [53]. We used the mean of all directional dyadic indexes plus one standard deviation (*M*+1 *SD*) to define the minimum edge value in these diagrams.

#### Measures of network analysis

By calculating multiple statistics of network analysis, we obtained the following measures for each chimpanzee: *strength* (an individual’s gregariousness), *eigenvector centrality* (a measure of how well connected an individual is within the network), *reach* (a measure of indirect connectedness, typically used to assess behavioral contagion), *clustering coefficient* (a measure of only how well the associates of one individual are outwardly connected to the network, typically used to assess an individual’s sociability), *affinity* (a measure of the average strength of one’s network neighbors, typically used to assess the gregariousness of one’s close associates), and the population means and standard deviations for all of these measures. These measures were calculated for symmetric matrixes (allogrooming and agonism) using each individual’s mean of actor and reactor values. These analyses are commonly calculated to gather information on networks with more than five individuals [53].

#### Matrix correlations

We conducted a QAP correlation analysis in UCINET to test for matrix correlations between the observed allogrooming, proximity, and agonistic data. This analysis takes in square matrixes and computes the correlation between the scores of each dyad across all matrixes. To construct a *p*-value, the analysis generates random matrixes (here 5,000; statistical power increases with more random matrixes) to compare those proportions of correlation against that of the real (or observed) data [56]. In order to test for correlations between the proximity (symmetric, one-sided) and the allogrooming and agonistic matrixes (asymmetric, two-sided) we transformed the proximity matrix into a two-sided *reciprocal* matrix to minimize the number of empty matrix values [56].

#### Community divisions by modularity

We conducted community division by modularity analyses in SOCPROG to test for significant subgroupings within the population. In this analysis, each individual is assigned a cluster, and each individual’s eigenvector (eig.) is provided, where values near zero indicate uncertainty in cluster assignment [53]. These analyses can be conducted with three or more individuals and are also accompanied by a population modularity value (Q). Population modularity values greater than approximately 0.30 indicate significant community structure [57]

#### Tests for preferred and avoided relationships

Tests for preferred and avoided relationships can *only* be conducted on associative measures; therefore, we conducted these tests using proximity data in SOCPROG. This analysis generates random association matrixes (here, 1000 times and within samples; statistical power increases with more random matrixes) for a given set of individuals (three or more) to test against the real (or observed) data. For the null hypothesis (i.e., that the variation in the real data is no greater than that of the randomly generated data) to be rejected, the pattern of observed dyadic association should be exceedingly different from the distribution of the random association indexes. This test produces a one-sided *p*-value based on the number of randomly generated matrixes with a higher standard deviation than that of the observed data, as well as significantly preferred dyads (a significantly high association index relative to other dyads in the group), significantly avoided dyads (a significantly low association index relative to other dyads in the group), and dyadic *p*-values that represent the percent of random association indexes that are less than the real association index for that dyad (i.e., real association index > 95% of random association indexes, *p* = 0.05). This analysis can only be conducted with association data (proximity) and cannot be conducted with interaction data (allogrooming or agonism) [53].

#### Tests for reciprocity and unidirectionality

We conducted tests for reciprocity or unidirectionality to better understand the relative direction of asymmetric behaviors within the population. These analyses investigate the hypothesis that asymmetric behaviors (allogrooming and agonism) are exhibited reciprocally among two or more individuals (e.g., the rate of interaction individual A directed towards B is correlated with the rate of interaction B directed towards A). If there is no correlation between the matrix and its transpose, the measure is said to be unidirectional. In SOCPROG we used Mantel *Z*-tests to examine absolute reciprocity (each dyad’s proportion of interaction compared to all other dyads), and the *Kr*-test to assess relative reciprocity (each dyad’s proportion of interactions compared to other dyads with the same actor) [52–53, 58].

#### Dominance linearity and ranks

Following conventional methods of analyzing dominance hierarchies within primate populations, we coded agonistic interactions in an actor-recipient dichotomous fashion. However, we observed a limited amount of agonism through our focal and scan sampling (see Results) and did not observe reciprocal contact aggression (i.e., fights). With the observations we collected, we characterized aggressive actors to have “won” the interaction, while aggressed-upon recipients were said to have “lost” the interaction. To incorporate as many interactions as possible in our analyses, we added submissive behaviors to our dominance analyses. Because submissive behaviors are assumed to be directed from lower to higher ranking individuals [59], we reverse-coded submissive behaviors so that the submitting actor was said to have “lost” to the recipient, or “winner” of the submission.

To investigate the degree of linearity within this population’s agonistic data, we conducted de Vries’ test for linearity [60]. This test derives the certainty (*h*’) of dominant individuals always acting in agonistic interactions over a recipient (or subordinate) individuals in a population of three or more individuals. This analysis evaluates the null hypothesis that these interactions are random and generates a *p*-value by testing the real data against a given number of random permutations (here, 1000; statistical power increases with more random permutations). Dominance interactions are said to be linear where *h*’ reaches or exceeds 0.90 [53].

We derived each chimpanzee’s dominance rank through multiple conventional methods in SOCPROG. *Modified David’s scores* derive a dominance index from count data for each individual so that those typically “dominating” have a large positive score, and those that are typically “dominated” have large negative scores [46]. *Brown’s ranking method* minimizes the proportion of dyadic interactions where any lower ranking individual “wins” an interaction [48]. de Vries’ *I & SI method* minimizes the sum of rank differences between inconsistently ranked individuals [47]. To assemble dominance hierarchies based on high-middle-low classifications of dominance positions [30] we coupled agonistic community division by modularity results with modified David’s scores [46] to assign each individual to a *hierarchically ranked cluster*. These tests derive individual dominance ranks relative to the ranks of other individuals within groups of three or more. Caution is urged when deciding which dominance ranking method is best for any given population, and authors should refer to the cited literature above for detailed discussions of these analyses [30, 36–48, 53].

## Results

We collected 6405 minutes of focal follow data (915 min/chimpanzee, 61 follows/chimpanzee) and 3122 instantaneous scan samples. Of these data, we observed 668 minutes of affiliation (213 min of allogrooming; see S1 Table) during focal-animal sampling and 55 aggressive interactions (29 displaces, 10 threats, 16 other agonism; see S2 Table) across focal-animal and scan samples. During instantaneous scan sampling, we observed a focal individual to be in proximity to at least one other chimpanzee 1578 times across all scans (see S3 Table). We calculated interobserver reliability between J.A.F. and an independent coder using Cohen’s kappa for nominal variables [61]. Reliability exceeded 85% agreement across all observations: focal sampling (κ = 0.88) and scan samples (κ = 0.93), as well as within proximate (κ = 0.93), affiliative (κ = 0.96), and aggressive (κ = 1.00) contexts. One hundred percent agreement (κ = 1.00) was achieved on individual chimpanzee identification.

### Dyadic indexes

**Fig 1:**
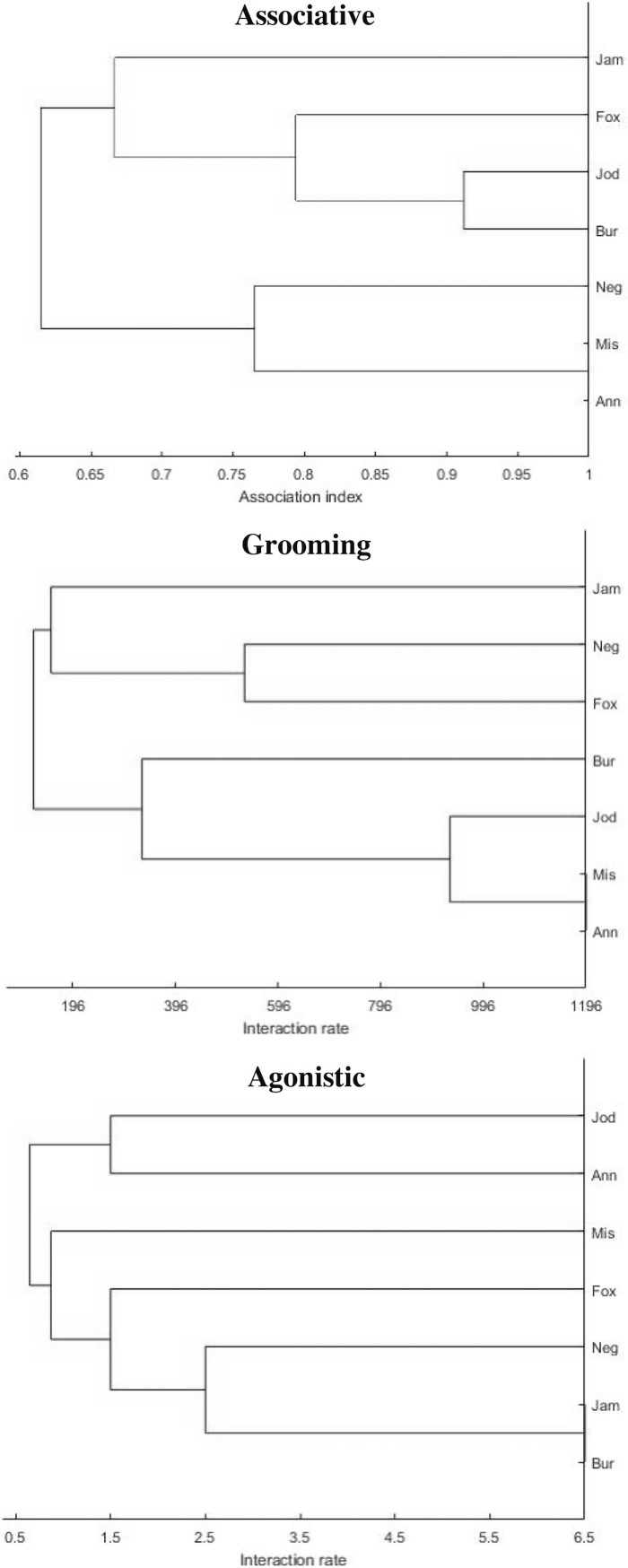
Associative, grooming, and agonistic hierarchical cluster analyses. These analyses were constructed from dyadic indexes in SOCPROG [52]. Associative CCC = 0.76; grooming CCC = 0.91; agonistic CCC = 0.84.

**Fig 2:**
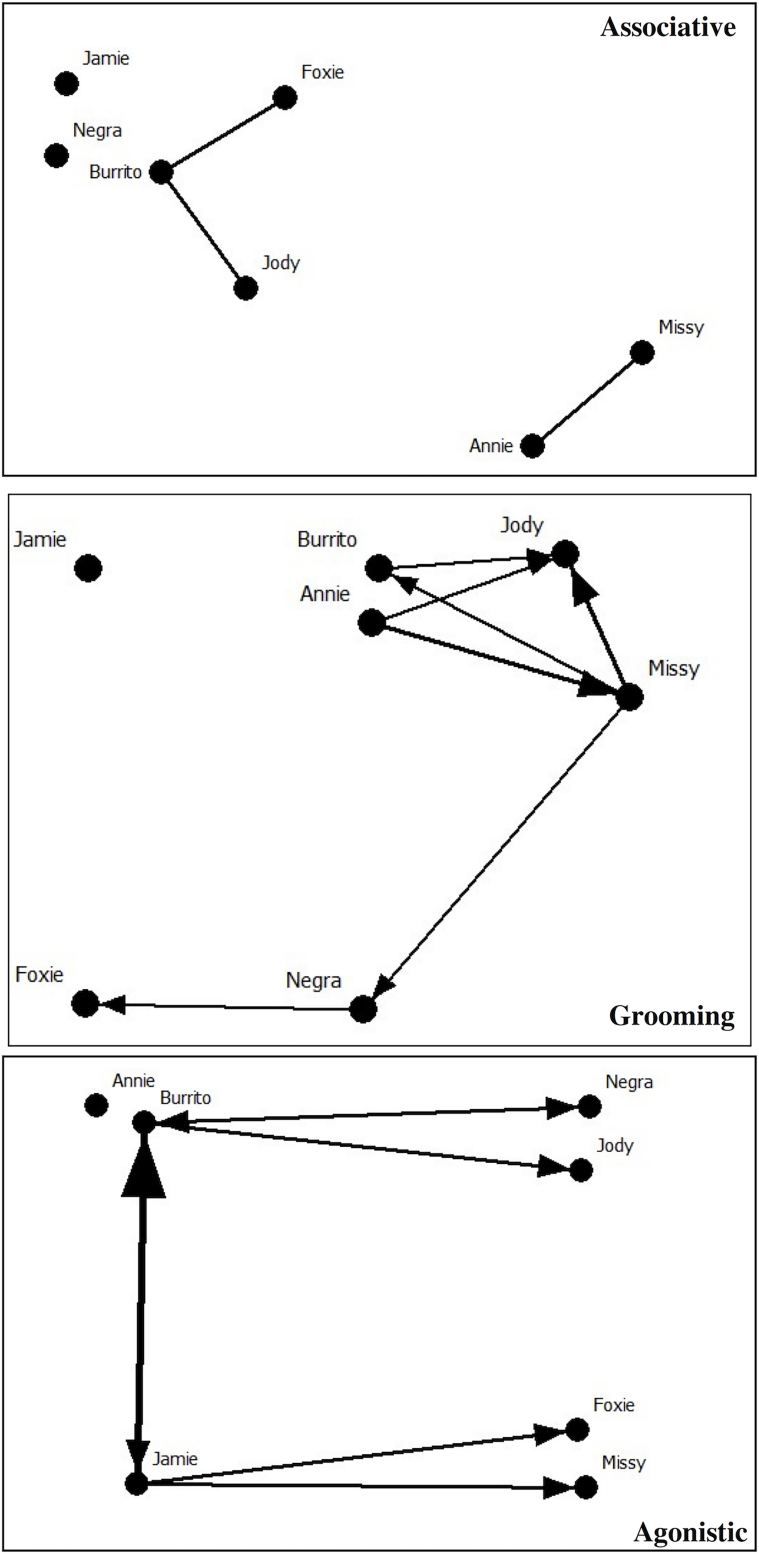
Associative, grooming, and agonistic principal coordinate analyses. The principal coordinates format was used to construct an associative, grooming, and agonistic network diagram drawn in NetDraw [55] using dyadic indexes derived from SOCPROG [52]. Edges are defined by the mean of all dyadic association indexes plus one standard deviation (*M*+1 *SD*). The grooming and agonistic diagrams depict directional relationships with line and arrow width representing relationship value.

Across all chimpanzee dyads, *association* indexes averaged 0.70 ± 0.13, ranging between 1.00 (Mis/Ann) and 0.41 (Bur/Neg). Across all dyads in each direction, *allogrooming* durations averaged 301.40 ± 549.18 s, ranging between 2262 s (Ann/Mis) and 0.00 s (four dyads did not groom at all: Fox/Ann, Jam/Ann, Neg/Bur, Bur/Jam). Across all dyads in each direction, *agonism* occurred an average of 1.31 ± 1.87 times, ranging between 10 (Jam/Bur) and zero events; however, aggression in most dyads occurred once or not at all (*N* = 11 dyads). Figs 1 and 2 depict the hierarchical cluster and principal coordinate analyses for the associative, grooming, and agonistic networks.

### Community divisions by modularity

The *associative* community divisions by modularity analysis were not indicative of significant community structure (*Q* = 0.06) [57]. The *grooming* community divisions by modularity analysis indicated significant community structure (*Q* = 0.46). This analysis separated the population into two clusters: Cluster 1, Ann (eig. = 0.27), Bur (eig. = -0.55), Jod (eig. = 0.55), and Mis (eig. = -0.51); and Cluster 2, Fox (eig. = 0.24), Jam (eig. = -0.04), and Neg (eig. = 0.04). Coupling our infrequent observations of *agonistic* events and our study population’s modularity at the threshold of significance (*Q* = 0.33), we report our derived agonistic clusters but do not consider them to appropriately represent this population’s subgroupings. This analysis separated the population into three clusters: Cluster 1, Mis (eig. = -0.14); Cluster 2, Ann (eig. = -0.06) and Jod (eig. = -0.01); and Cluster 3, Bur (eig. = 0.69), Fox (eig. = -0.08), Jam (eig. = 0.70), and Neg (eig. = 0.13).

### Measures of network analysis

The results from the *associative* network analyses (Table 3) indicated Ann was most often in proximity to other individuals (greatest network strength), the most closely associated with other highly connected individuals (highest eigenvector centrality), and most indirectly connected with other individuals (furthest reach). The results also indicated that Neg was weakly connected to the population but well associated with other chimpanzees who were highly associated with the rest of the population (highest clustering coefficient and affinity).

**Table 3:** Associative, grooming, and agonistic measures of network analysis.

Measure	Chimpanzee	Strength	Eigenvector Centrality	Reach	Clustering Coefficient	Affinity
Proximity	Ann	4.50*	0.41*	18.36*	0.66	4.08
	Bur	4.12	0.38	17.13	0.69	4.16
	Fox	4.09	0.37	16.92	0.69	4.14
	Jam	3.74	0.35	15.80	0.71	4.20
	Jod	4.41	0.40	18.02	0.67	4.08
	Mis	4.32	0.39	17.73	0.67	4.10
	Neg	3.62	0.34	15.83	0.73*	4.25*
	Mean (± SD)	4.12 (± 0.33)	0.38 (± 0.03)	17.05 (± 1.12)	0.49 (± 0.02)	4.14 (±0.06)
Grooming	Ann	2055.00	0.49	6424694.25	0.83*	3126.37*
	Bur	1207.00	0.24	3193649.25	0.50	2645.94
	Fox	1060.50	0.12	1733100.00	0.17	1634.23
	Jam	593.50	0.09	1308072.00	0.37	2204.00
	Jod	2860.50	0.53	6502320.00	0.40	2273.14
	Mis	3420.00*	0.59*	6920119.50*	0.29	2023.43
	Neg	1462.50	0.22	3092588.00	0.25	2114.59
	Mean (± SD)	1808.43 (± 1023.20)	0.33 (± 0.20)	4167791.86 (± 2391989.30)	0.40 (± 0.22)	2288.81 (±477.10)
Agonism	Ann	4.00	0.16	41.75	0.35	10.44
	Bur	16.50*	0.62*	137.00	0.12	8.30
	Fox	5.00	0.26	59.75	0.55*	11.95
	Jam	12.50	0.56	138.25*	0.20	11.06
	Jod	5.50	0.22	55.50	0.21	10.09
	Mis	5.00	0.21	48.50	0.31	9.70
	Neg	6.50	0.34	86.25	0.47	13.27*
	Mean (± SD)	7.86 (± 4.74)	0.34 (± 0.02)	81.00 (± 41.11)	0.31 (± 0.15)	10.69 (± 1.61)

Network statistics calculated in SOCPROG for each measure and all chimpanzees.

* indicates the highest value in each column for each measure.

The results from the *grooming* network analyses (Table 3) indicated that Mis was the most involved in grooming (greatest network strength), the most closely associated with other highly connected individuals (highest eigenvector centrality), and the most indirectly connected with other individuals (furthest reach). The results also indicated that Ann was well associated with chimpanzees who were highly associated with the rest of the group, but was only weakly associated herself (highest clustering coefficient and affinity). Jam was the least involved in grooming, not well associated with other individuals, and had little indirect connectedness overall.

The results from the *agonistic* network analyses (Table 3) indicated that Bur was the most involved in agonism (most network strength) and involved in agonism with other relatively agonistic individuals (highest eigenvector centrality; i.e., Jam), whereas Jam was most indirectly connected to other individuals (furthest reach) and also considerably involved in agonism and involved with other agonistic individuals (i.e., Bur). Our results also indicated that Fox and Neg were well associated with chimpanzees who were relatively agonistic with the rest of the group but were only weakly agonistically connected themselves (highest clustering coefficient and affinity). Additionally, Ann, Fox, and Jod were least agonistically connected across all analyses.

### Matrix correlations

The QAP correlations resulted in three pair-wise statistics between the three matrixes. There was a significant weak, positive correlation between the allogrooming and proximity matrixes (*r* = 0.289, *p* = 0.015). There were no significant correlations between the allogrooming and agonistic (*r* = -0.059, *p* = 0.44) or agonistic and proximity matrixes (*r* = -0.124, *p* = 0.21).

### Preferred and avoided relationships

The test for preferred and avoided relationships from the associative data (proximity) was significant (*p* < 0.001); therefore, the null hypothesis was rejected. This test identified four dyads with significantly preferred relationships and three dyads with significantly avoided relationships (Table 4).

**Table 4:** Preferred and avoided relationships.

Dyad	Relationship	Association Index	p-value
Mis/Ann	Preferred	1.00	0.001
Jod/Bur	Preferred	0.91	0.005
Fox/Bur	Preferred	0.88	0.002
Neg/Mis	Preferred	0.79	0.01
Mis/Jod	Avoided	0.65	0.02
Bur/Ann	Avoided	0.62	0.004
Neg/Bur	Avoided	0.41	0.02

Results of preferred and avoided relationship analyses in SOCPROG through measures of proximity (associations).

### Tests for reciprocity or unidirectionality

Tests for reciprocity or unidirectionality for the allogrooming network found no significant reciprocity (Mantel *Z*-test, *p* = 0.95; Hemelrijk *Kr*-test, *p* = 0.61), indicating that the chimpanzees of this group groomed unidirectionally. However, these chimpanzees were significantly reciprocal in their agonistic interactions (Mantel *Z*-test, *p* = 0.049; Hemelrijk *Kr*-test, *p* = 0.03).

### Dominance hierarchy

de Vries’ test for dominance linearity [60] indicated the hierarchy of this population was not significantly linear (*h’* = 0.43, *p* = 0.38). This result indicates a non-linear hierarchy with inconsistencies between individual ranks. We calculated the dominance rank for each individual using multiple methods (Table 5). Notably, no two methods ranked the group in a similar fashion. Jam was consistently ranked as most dominant while Mis was consistently ranked least dominant, but all other individuals were ranked in various positions across the ranking methods.

**Table 5:** Individual chimpanzee dominance ranks using multiple measures.

ID	Modified David's Score	Brown	I & SI	Hierarchical Clusters
Jam	5.71	1	1	1
Ann	1.75	5 / 6	4	2
Bur	1.71	3	2	1
Fox	1.35	2	6	1
Jod	-0.67	4	3	2
Neg	-1.28	6 / 5	5	1
Mis	-8.58	7	7	3

The results of the dominance hierarchy analyses in SOCPROG. Brown’s [48] method for deriving dominance hierarchy ranked Neg and Ann in reciprocal positions. Therefore, both rankings are listed.

## Discussion

This investigation successfully examined and described the social structure and dominance hierarchy of seven chimpanzees at Chimpanzee Sanctuary Northwest. Our results indicated that three individuals were most central and highly connected in the grooming network, while two others had little connection. Through agonistic networks, we found that group members reciprocally exhibited agonism, and the group’s dominance hierarchy was statistically non-linear. One chimpanzee emerged as the most dominant through agonism but was least connected to other group members across affiliative networks. Our results indicate that the conventional methods used to calculate individuals’ dominance rank may be inadequate to wholly depict the group’s social relationships in this captive sanctuary population. Our results also have an applied component that can aid sanctuary staff in a variety of ways to best ensure a positive trend in the improvement of captive chimpanzee welfare.

### Grooming and proximity

The affiliative and associative analyses were informative regarding each chimpanzee’s position within the social network at CSNW. Based on their association index of 1.00, it is apparent that Mis/Ann have a strong and significantly preferred relationship. Additionally, Ann spends more time in proximity to other individuals than she spends engaged in grooming, as indicated by her strong associative but weak grooming strengths. However, Ann and Mis together, remain central to the group through their relationship with one another (Mis through the strong associative relationships of Ann and Ann through the strong grooming relationships of Mis). These two chimpanzees are only connected to each other in the associative network, but are better connected to the rest of the network through measures of allogrooming (Fig 2).

Within the grooming network, Jod maintains a noteworthy central position within the population. Similar to Ann, Jod demonstrated a preference for associating with others more so than grooming, evident through her strong associative strength, centrality, and reach, but moderately weak grooming network measures. The grooming divisions by modularity indicated that Mis/Ann/Jod/Bur comprised a significant subgroup within this population. These four individuals were the most active in grooming and most frequently groomed one another; notably, Jod received the greatest proportion of grooming within the group. However, Jod/Mis and Bur/Ann had significant avoided relationships through measures of proximity. This result might indicate the Jod/Mis and Bur/Ann dyads spend little time near one another except while grooming.

By generalizing across the allogrooming and proximity measures, the results from the two measures demonstrate that Neg was only connected to the group through her grooming relationships with Mis and Fox, but an isolate in the associative network. And, Fox was most notably connected to the group through her significantly preferred relationship with Bur. Consistent across both measures, Jam was an isolate of the group with no notable connections to any other chimpanzee. It is this isolated position of Jam in affiliative networks that calls into question her position as the most dominant individual through measures of agonism.

Appropriately measuring and representing non-human primate social relationships is challenging [16–17], however, by utilizing two common behavioral measures (allogrooming and proximity) to gauge such relationships, we constructed a broader understanding of this particular group’s overall affiliative network. The significant (yet, weakly positive) correlation of the proximity and allogrooming matrixes indicated that this group’s affiliative interactions and dyadic relationships were relatively consistent across measures. We posit that the observed patterns of grooming might indicate strong affiliative bonds [18–19], whereas patterns of non-contact association might indicate dyadic social tolerance and be less indicative of strong affiliative bonds. Relative to other settings, captive individuals have less choice about who they spend their time near. SNA allowed us to consider different types of affiliative measures (allogrooming and proximity) while avoiding over-simplifying a complex network or overlooking individual network positions. Indeed, SNA can be more useful than conventional statistical analyses to address the degree and strength of social relationships at different levels (e.g., [10, 13, 37, 53]). Further, given the dyadic nature of social interaction data (see S1–S3 Tables) independence and normal distributions cannot be assumed and therefore conventional inferential statistics (e.g., ANOVA, Kruskal-Wallis, or Chi-squared Goodness of Fit or Test of Independence) are not appropriate for such investigations [53]. SNA allows for data to represent a wide array of variability in social relationships (e.g., Neg-Bur vs. Ann-Mis allogrooming) as they occur in nature to compute any number of relevant statistics. Although inferential statistics are useful to analyze the effects of individual characteristics on a given variable, such analyses are unable to reveal the effects of individuals on the entire network structure, depict a holistic representation of individual relationships, or lend themselves to a greater understanding of how dyadic relationships scale in relation to the whole network [10].

### Agonism and dominance

Notably, Jam was only connected to the group through agonism and was peripheral in the other networks. She and Bur were the most frequent actors within aggressive contexts (typically directed at one another). Similarly, although Jod and Mis were well connected in affiliative networks, they were most notably the recipients in agonistic encounters rather than the actors. Agonism was directed towards Fox by Jam, whereas Neg and Bur engaged in seemingly reciprocal agonism. Ann, who was most central in the associative networks, was an isolate in the agonistic network. Finally, we observed the members of this chimpanzee group exchange agonism in a reciprocal manner, and we found that the dominance hierarchy was non-linear.

Although the dominance hierarchy was statistically non-linear, the conventional methods [30, 46–48] we used to assess dominance reported the hierarchy in a mutually exclusive, step-wise fashion; however, linearity should not be presumed simply because individuals are ranked in such a way (Table 5). These statistical measures are typically defined using each individual’s proportional rate of acting-over-receiving aggression. In other words, if chimpanzee Y directs aggression towards other individuals more so than others direct aggression towards chimpanzee Y, chimpanzee Y is calculated as more dominant. Because we used agonism for these dominance analyses, the resulting dominance hierarchies were defined through the individuals’ aggressiveness.

Interestingly, the dominance ranks we calculated by each method differed for most chimpanzee, but notably, all analyses assigned Jam as the most dominant individual. This result is possibly biased towards individual proportions of actor/recipient paradigms: because Jam directs so much aggression towards other individuals but is not acted upon by many other individuals, the dominance rank analyses calculate that she is most “dominant” because she is “winning” (or acting) in more agonistic interactions than she is “losing” (or receiving). Considering the isolate position of Jam in the affiliation networks, it is not obvious that she holds a significant place in the overall social network (e.g., [40]). Because of the limitations in the computational methods used to derive dominance hierarchies, the overall lack of observed agonism within this particular group, and the biases towards analyzing only one behavioral marker of dominance (namely, aggression), we do not find the derived dominance hierarchies in Table 5 to appropriately reflect the social dynamics and relationships within this population. Therefore, we find it more appropriate to conclude that Jam, in particular, engages in agonistic interactions more so than the other chimpanzees rather than labeling her as the most dominant.

Additionally, the agonism analyses of this group do not generate much clarity regarding dominance, and we believe this to be mainly because we observed little agonism during the study period (fewer than 2 events/day) [31]. This was not simply a limitation of this specific investigation: other authors have also noted the scarcity of observing agonism in captivity [43–44], specifically between female chimpanzees [62]. Even free-ranging studies have focused on male-male agonism because females are involved so sporadically (partially because of typical ranging patterns) [63]. Only because we used multiple social network measures (proximity, grooming, and agonism) can the results of the dominance rank analyses be questioned. This highlights the importance for future investigators to consider behavioral data collection methods that ensure the observation of a broader range of possible social events (e.g., all-occurrence). Perhaps more pointed dominance analyses like Elo-ratings [64], PERC [65], or ADAGIO [32] could be conducted with a larger set of agonistic data to derive an appropriate hierarchy that offers a more comprehensive understanding of the complex social dynamics of these chimpanzees.

### Captive management

With our results, we aim to help the managers of CSNW provide the best possible care and to continue to improve this groups’ overall welfare. We conclude from the results of our affiliative analyses that Mis, Ann, and Jod are most central with the highest strength and furthest reach. Therefore, these individuals may significantly influence group stability and aggression mitigation [37]. With this knowledge, sanctuary staff can best prepare for attrition of these individuals by introducing chimpanzees that are highly sociable or by temporarily separating clusters of individuals that were only connected by these central group members. This might diminish any adverse effects on group stability and probable increased aggression. Furthermore, sanctuary staff may plan to introduce incoming chimpanzees to these three most central individuals to mitigate aggression and increase cohesion upon introduction to the entire group.

Additionally, SNA can increase our understanding of individual- and group-level social relationships, render visible social network diagrams, and contribute to improved management and welfare protocols. In captivity, it is difficult to replicate the opportunity for chimpanzee social groups to engage in the fission-fusion dynamics of their wild counterparts; yet, the ultimate success of a captive environment is often judged by its replication of the wild setting [66–67]. Therefore, future research could investigate how to use the tools provided by SNA (e.g., community divisions of modularity, subgroup analyses, individual eigenvector centrality, and comparison of multiple measures of sociality) to suggest methods for the successful implementation of fission-fusion structures that better reflect the social life and choices of wild chimpanzees. Achieving this would be beneficial in propelling social agency and autonomy at any captive great ape facility [67].

Finally, our analyses provided evidence to suggest that Jam was relatively agonistic yet an isolate in affiliative networks. This juxtaposition might question if she is best suited for this specific group and her effect on other group members. However, because the overall rates of agonism within this group were infrequent and the group is relatively small, we do not see a need for Jam’s separation. Our analyses derived significant subgroupings to consider when making such captive management decisions, which can aid in addressing future questions of group composition at CSNW and other chimpanzee sanctuaries. In order to increase the reciprocity of affiliative behaviors and decrease deleterious aggression between many individuals, identifying subgroups through SNA could suggest where the group’s structure could be modified/restructured to increase group cohesion.

Together with the results of other authors [2, 10, 13, 36–37, 41], the methods and analyses used here to model patterns of captive chimpanzee sociality can be readily applied in other captive settings. In this sanctuary setting, SNA was a novel and informative tool to address many questions regarding group composition, individual network position, and group-level social systems. These results contribute to the continued discussion of best practices in non-human primate captive management and improved captive welfare at a global level beyond the five freedoms [33]. We recommend the utility of SNA to those seeking nuanced analyses of social group composition.

## Supporting information

S1 Table Allogrooming matrix. Total observed durations of allogrooming (in seconds) for each chimpanzee dyad are reported in an asymmetric (actor-reactor) matrix.
(PDF)

S2 Table Agonism matrix. Total observed occurrences of agonism for each chimpanzee dyad are reported in an asymmetric (actor-reactor) matrix.
(PDF)

S3 Table Association indexes (proximity) matrix. Calculated simple ratio association indexes for each chimpanzee dyad are reported in a symmetric (unidirectional) matrix.
(PDF)
