# Supplementary material for: Social network and dominance hierarchy analyses at Chimpanzee Sanctuary Northwest
Source: PLoS One. 2018 Feb 14;13(2):e0191898. doi: 10.1371/journal.pone.0191898 (PMC5812591; doi:10.1371/journal.pone.0191898)
Supplement: S1 Table — Total observed durations of allogrooming (in seconds) for each chimpanzee dyad are reported in an asymmetric (actor-reactor) matrix. (PDF) [file pone.0191898.s001.pdf]

Actor \ Reactor	Ann	Bur	Fox	Jam	Jod	Mis	Neg
**Ann**	0	45	0	0	1567	2262	16
**Bur**	90	0	394	0	1197	0	0
**Fox**	0	36	0	97	0	119	262
**Jam**	0	0	169	0	127	0	349
**Jod**	0	0	13	110	0	0	471
**Mis**	130	652	232	335	2159	0	951
**Neg**	0	0	799	0	77	0	0
