# Supplementary material for: Social network and dominance hierarchy analyses at Chimpanzee Sanctuary Northwest
Source: PLoS One. 2018 Feb 14;13(2):e0191898. doi: 10.1371/journal.pone.0191898 (PMC5812591; doi:10.1371/journal.pone.0191898)
Supplement: S2 Table — Total observed occurrences of agonism for each chimpanzee dyad are reported in an asymmetric (actor-reactor) matrix. (PDF) [file pone.0191898.s002.pdf]

Reactor

Actor	Ann	Bur	Fox	Jam	Jod	Mis	Neg
**Ann**	0	1	0	1	1	1	0
**Bur**	2	0	0	3	4	2	5
**Fox**	0	2	0	2	0	1	0
**Jam**	0	10	4	0	0	3	2
**Jod**	2	1	0	0	0	1	1
**Mis**	0	0	0	0	1	0	0
**Neg**	0	3	1	0	0	1	0
