# Supplementary material for: Social network and dominance hierarchy analyses at Chimpanzee Sanctuary Northwest
Source: PLoS One. 2018 Feb 14;13(2):e0191898. doi: 10.1371/journal.pone.0191898 (PMC5812591; doi:10.1371/journal.pone.0191898)
Supplement: S3 Table — Calculated simple ratio association indexes for each chimpanzee dyad are reported in a symmetric (unidirectional) matrix. (PDF) [file pone.0191898.s003.pdf]

	**Ann**	**Bur**	**Fox**	**Jam**	**Jod**	**Mis**	**Neg**
**Ann**							
**Bur**	0.62						
**Fox**	0.68	0.88					
**Jam**	0.65	0.65	0.76				
**Jod**	0.82	0.91	0.71	0.59			
**Mis**	1.00	0.65	0.59	0.65	0.65		
**Neg**	0.74	0.41	0.47	0.74	0.74	0.79	
